# Associations between smoking and clinical outcomes after total hip and knee arthroplasty: A systematic review and meta-analysis

**DOI:** 10.3389/fsurg.2022.970537

**Published:** 2022-11-02

**Authors:** Chen Yue, Guofeng Cui, Maoxiao Ma, Yanfeng Tang, Hongjun Li, Youwen Liu, Xue Zhang

**Affiliations:** ^1^ Department of Orthopedic Surgery, Luoyang Orthopedic Hospital of Henan Province. Orthopedic Hospital of Henan Province, Luoyang, China; ^2^ Department of Orthopedic Surgery, Luoyang Central Hospital Affiliated to Zhengzhou University, Luoyang, China

**Keywords:** smoking, clinical outcomes, THA, TKA, meta-analysis

## Abstract

**Background:**

Smoking increases risk of several complications after total hip or knee arthroplasty (THA/TKA), so we systematically reviewed and meta-analyzed the literature to take into account all relevant evidence, particularly studies published since 2010.

**Methods:**

The PubMed, Ovid Embase, Web of Science, and EBSCOHost databases were searched and studies were selected and analyzed according to MOOSE recommendations. Methodological quality of included studies was assessed using the Newcastle-Ottawa Scale. Data were qualitatively synthesized or meta-analyzed using a random-effects model.

**Results:**

A total of 40 studies involving 3,037,683 cases were included. Qualitative analysis suggested that smoking is associated with worse patient-reported outcomes within one year after surgery, and meta-analysis showed that smoking significantly increased risk of the following outcomes: total complications (*OR* 1.41, 95% *CI* 1.01–1.98), wound complications (*OR* 1.77, 95% *CI* 1.50–2.10), prosthetic joint infection (*OR* 1.84, 95% *CI* 1.52–2.24), aseptic loosening (*OR* 1.62, 95% *CI* 1.12–2.34), revision (*OR* 2.12, 95% *CI* 1.46–3.08), cardiac arrest (*OR* 4.90, 95% *CI* 2.26–10.60), cerebrovascular accident (*OR* 2.22, 95% *CI* 1.01–4.85), pneumonia (*OR* 2.35, 95% *CI* 1.17–4.74), acute renal insufficiency (*OR* 2.01, 95% *CI* 1.48–2.73), sepsis (*OR* 4.35, 95% *CI* 1.35–14.00), inpatient mortality (*OR* 12.37, 95% *CI* 4.46–34.28), and persistent opioid consumption (OR 1.64, 95% CI 1.39–1.92).

**Conclusion:**

Smoking patients undergoing THA and TKA are at increased risk of numerous complications, inpatient mortality, persistent opioid consumption, and worse 1-year patient-reported outcomes. Pre-surgical protocols for these outcomes should give special consideration to smoking patients.

Smoking increases the risk of various diseases, including cancer, stroke, as well as diseases affecting the heart, lungs and peripheral vasculature (1–5). A global epidemiological survey of 3 billion individuals indicated the rate of smoking to be 48.6% in men and 11.3% in women (6). As a result, smoking is a leading cause of preventable premature mortality worldwide (3). Preoperative smoking is common among patients undergoing elective surgery, and it increases risk of several postoperative complications, such as wound complications, pulmonary complications, and general infections (7, 8).

Total hip and knee arthroplasty (THA/TKA) are effective surgical procedures to improve function and reduce pain in patients with severe hip and knee joint disease. By 2030, 572,000 THA procedures and 3.48 million TKA procedures will likely be performed in the United States alone (9). It is reported that up to one-third of patients may experience various complications after THA/TKA, which has a negative impact on the rehabilitation and satisfaction of patients (9–13). National databases from several countries indicate a smoking prevalence of 10%–40% among THA/TKA patients (10–13). Numerous studies have examined the impact of smoking on postsurgical outcomes in these patients. Certain studies have concluded that smoking increases risk of systemic complications (14, 15), surgical complications (16–18), mortality (19), or readmission (20), or that it is associated with worse patient-reported outcomes (12). However, other studies have failed to find a correlation between smoking and these complications or poor outcomes (11, 21–24). A systematic review covering literature published up to 2010 concluded that smoking is associated with significantly higher risk of any postoperative complication and mortality following THA or TKA (25).

Since that review, more than 30 studies have been published that broadened and deepened our understanding of how smoking affects outcomes after THA and TKA. We therefore comprehensively assessed relevant studies published since 2000 through 2022, and we meta-analyzed or qualitatively synthesized data on 21 indices in seven outcomes: total complications, surgical complications, systemic complications, mortality, readmission, opioid consumption, and patient-reported outcomes.

## Methods

This review was reported in line with MOOSE guidelines (26, 27). Two authors independently searched the databases of PubMed, Ovid Embase, Web of Science, and EBSCOHost from January 2000 to August 2022 using comprehensive search strategies (Search strategies applied to each database could be found in Supplementary Figure S1A–D). Reference lists of relevant articles were also reviewed to identify additional eligible studies. Language experts were contacted for translation of articles not written in English.

### Study selection

A study was considered eligible for inclusion if it (1) was a cohort study based on smoking status, or a case-control study that considered smoking as a possible risk factor; and (2) it reported data about the potential association of smoking with at least one of the outcomes of interest following THA or TKA. A study was excluded if it (1) was based on a cross-sectional questionnaire, pilot study, case report, case series report, or brief report; (2) was published only as an abstract; or (3) involved revision surgery, hemiarthroplasty, unicompartment knee arthroplasty, or arthroscopic surgery.

All retrieved studies were imported into Endnote × 7 (Thomson Scientific, Stamford, Connecticut, United States). The same two authors who had searched the databases independently excluded irrelevant studies based on titles and abstracts. These two authors then read the full text of the remaining articles to produce a final list of studies. Any discrepancies between the two authors were resolved through discussion with a third author.

### Data extraction

The same two authors independently extracted the following data from each eligible study: first author's name, country, publication year, surgery type, sample size, study type, and follow-up time. Surgical complications included wound complications (any wound problems such as superficial/deep infection, exudation, or hematoma), prosthetic joint infection, aseptic loosening, dislocation, and revision. Systemic complications included circulatory complications, respiratory complications, urinary complications, venous thromboembolism, and sepsis. Patient-reported outcomes were evaluated based on various scales such as Harris Hip Scores and Oxford Hip/Knee Score.

### Assessment of study quality and evidence quality

The same two authors who searched the databases also independently evaluated the methodological quality of each study based on the Newcastle-Ottawa Scale (NOS), which is a widely used quality evaluation tool for observational studies (28). The maximum total NOS score is 9, and only studies scoring at least 5 were included in the meta-analysis. The quality of evidence for each outcome was evaluated according to the Grading of Recommendations Assessment, Development, and Evaluation (GRADE) system (29, 30).

### Statistical analysis

Data on patient-reported outcomes were synthesized qualitatively because the evaluation methods varied substantially across studies. Other outcomes were meta-analyzed using random-effects model and displayed as forest plots using STATA Release 14 (Stata Corp, College Station, Texas, United States). All variables in this study were dichotomous, and pooled risk estimates were expressed as odds ratios (*ORs*) with 95% confidence intervals (95% *CIs*). A 2-tailed P value < 0.05 was defined as statistically significant.

Heterogeneity across studies was assessed using the *I*^2^ test and was considered substantial if *I*^2^ > 50% (31). In meta-analyses involving at least 10 studies and substantial heterogeneity, meta-regression analyses were conducted based on publication year, country, follow-up time, surgery type, or NOS score in order to identify potential sources of heterogeneity (32). Subgroup analysis based on the study type (cohort vs. case-control) was also conducted to detect any influence on outcomes.

We planned to perform some or all of the following sensitivity analyses for each outcome: (1) exclude studies involving fewer than 500 or 1,000 patients, (2) exclude studies involving more than 100,000 or 1,000,000 patients, (3) exclude studies involving fewer than 100 events, (4) exclude studies scoring no more than 6 on the NOS, and (5) exclude studies flagged as causing heterogeneity after running the “hetred” command in STATA.

## Results

### Search results and characteristics of included studies

**Figure 1:**
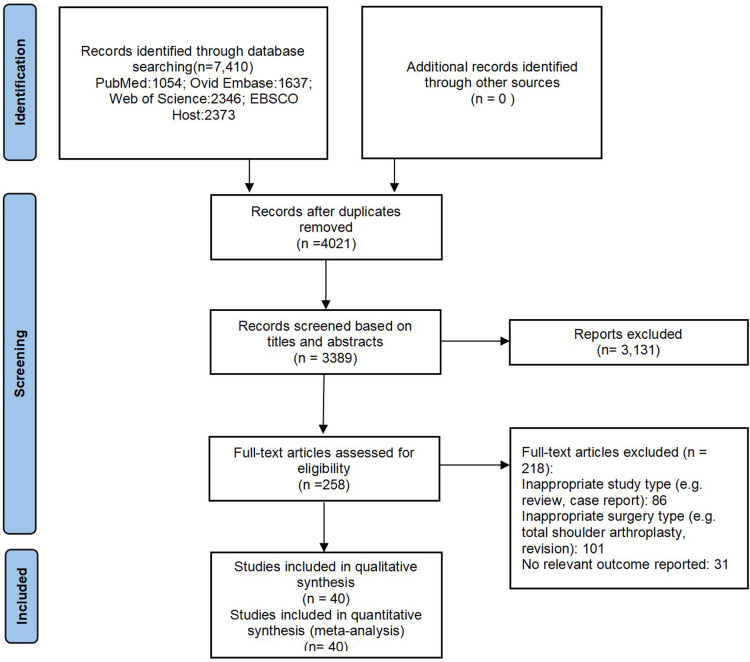
Flowchart of study identification, screening, and selection.

A total of 7,410 records were identified and no additional records were found through manual searching of references. An initial screening removed 4,021 duplicate records, and another 3,131 records were excluded as irrelevant after reading titles and abstracts. A further 218 records were removed because they failed to satisfy selection criteria. In the end, 40 studies involving 3,037,683 cases were included for meta-analysis (10–16, 18–24, 33–58). The details of study identification, inclusion, and exclusion are shown in Figure 1. The included 40 studies contained 20 cohort studies (10, 11, 14, 15, 19, 20, 23, 40, 41, 43, 45–48, 51–53, 55–57) and 20 case-control studies (12, 13, 16, 18, 21, 22, 24 33–39, 42, 44, 49, 50, 54, 58),. Nearly all studies had been published since 2005, including 35 since 2010 and 20 since 2017. Table 1 provides a detailed description of the study characteristics.

**Table 1:** Description of studies and participants.

Reference	Country	Surgery	Sample size	Follow-up	Comparison	Study type	NOS
Ali Vial (2020) (33)	UK	THA/TKA	197	3 days	Smoker; Nonsmoker	CC, S	7
Anderson (2017) (34)	United States	THA/TKA	66,985	At least 2 years	Smoker; Nonsmoker	CC, M*	8
Baier (2019) (16)	Germany	TKA	2,439	1 year	Smoker; Nonsmoker	CC, S	7
Bedard (2018) (35)	United States	TKA	35,894	2 years	Smoker; Nonsmoker	CC, M*	8
Bohl (2017) (36)	United States	THA/TKA	171,200	30 days	Current smoker; Nonsmoker	CC, M*	8
Bohl (2016) (37)	United States	THA/TKA	117,935	30 days	Current smoker; Nonsmoker	CC, M*	8
Burn (2019) (11)	UK	THA/TKA	21,221	10 years	Current smoker; Former smoker; Nonsmoker	CO, M*	7
Chrastil (2015) (38)	United States	THA/TKA	13,272	2 years	Smoker; Nonsmoker	CC, M*	7
Debbi (2019) (19)	United States	THA	317,230	Hospitalization	Smoker; Nonsmoker	CO, M*	7
Debreuve-Theresette (2015) (39)	France	THA/TKA	135	1 year	Smoker; Nonsmoker	CC, S	8
Duchman (2015) (10)	United States	THA/TKA	78,191	30 days	Current smoker; Former smoker; Nonsmoker	CO, M*	8
Etcheson (2018) (40)	United States	THA	248	90 days	Smoker; Nonsmoker	CO, S	8
Gonzalez (2018) (41)	Switzerland	THA/TKA	8,559	Median 67 months	Current smoker; Former smoker; Nonsmoker	CO, S	8
Grammatico-Guillon (2015) (42)	France	THA/TKA	32,678	At least 1year	Smoker; Nonsmoker	CC, M	6
Halawi (2018) (43)	United States	THA/TKA	711	1 year	Smoker; Nonsmoker	CO, S	7
Hassan (2015) (22)	Denmark	THA	586	1 year	Smoker; Nonsmoker	CC, S	6
Hassan (2015) (21)	Denmark	TKA	647	1 year	Smoker; Nonsmoker	CC, S	6
Hesseling (12)	Netherlands	THA	6,030	1 year	Smoker; Nonsmoker	CC, M*	7
Jørgensen (2018) (44)	Denmark	THA/TKA	8,975	1 year	Smoker; Nonsmoker	CC, S	7
Kapadia (2014) (45)	United States	THA	330	Mean 51 months	Smoker; Nonsmoker	CO, S	7
Kapadia (2012) (46)	United States	TKA	621	Mean 47 months	Smoker; Nonsmoker	CO, S	7
Khan (2009) (47)	UK	THA	1,767	Maximum 5 years	Current smoker; Former smoker; Nonsmoker	CO, S	6
Kim SC (2017) (13)	United States	THA/TKA	57,545	1 year	Smoker; Nonsmoker	CC, M*	7
Lübbeke (48)	United States	THA	1,964	Mean 6.9 years	Smoker; Nonsmoker	CO, S	6
Malik (2004) (23)	UK	THA	225	Unclear	Current smoker; Former smoker; Nonsmoker	CO, S	7
Matharu (2019) (15)	UK	THA/TKA	117,024	Various^†^	Current smoker; Former smoker; Nonsmoker	CO, M*	7
Møller (2003) (14)	Denmark	THA/TKA	811	4 weeks	Current smoker; Nonsmoker	CO, D	7
Nwachukwu (2015) (49)	United States	TKA	436	Unclear	Current smoker; Nonsmoker	CC, S	5
Peters (2020) (50)	Netherlands	THA	101,397	Median 4.9 years (1 to 12 years)	Smoker; Nonsmoker	CC, M*	8
Rajaee (2020) (51)	United States	TKA	1,801,705	Hospitalization	Smoker; Nonsmoker	CO,M*	7
Sadr Azodi (2006) (53)	Sweden	THA	3,304	2 months	Current smoker; Former smoker; Nonsmoker	CO, M*	7
Sadr Azodi (2008) (52)	Sweden	THA	2,106	Mean 2 years	Current smoker; Former smoker; Nonsmoker	CO, M*	7
Sahota (2017) (20)	United States	THA/TKA	2,502	30 days	Current smoker; Nonsmoker	CO, M*	8
Sikora-Klak (2017) (54)	United States	THA/TKA	2,907	90 days	Smoker; Nonsmoker	CC, S	8
Singh (2011) (55)	United States	THA/TKA	33,336	30 days	Current smoker; Former smoker; Nonsmoker	CO, M*	7
Singh (2015) (56)	United States	THA/TKA	7,926	2 years	Current smoker; Nonsmoker	CO, S	8
Suzuki (2011) (24)	Japan	TKA	2,022	Median 42 months	Smoker; Nonsmoker	CC, S	7
Tischler (2017) (57)	United States	THA/TKA	15,264	90 days	Current smoker; Former smoker; Nonsmoker	CO, S	8
Wu (2014) (18)	China	THA/TKA	437	Unclear	Smoker; Nonsmoker	CC, S	6
Xu (2019) (58)	China	THA/TKA	921	At least 1year	Smoker; Nonsmoker	CC, S	8

Co, Cohort study; CC, case-control study; S, single-center; D, double-center; M, multi-center; NOS, newcastle-ottawa scale; THA, total hip arthroplasty; TKA, total knee arthroplasty.

* National/population-based database.

^†^ 6 Months: complications and patient-report outcomes; 1 year: opioid consumption, readmission, and mortality; 20 years: revision.

### Outcomes

## Total complications

Total complications were investigated in eight cohort studies involving 552,553 patients (10, 14, 15, 19, 20, 45, 53, 55). Meta-analysis showed that smoking patients were at higher risk of total complications after THA and TKA than non-smoking patients (*OR* 1.41, 95% *CI* 1.01–1.98; *I*^2 ^= 99%; Table 2).

**Table 2:** Outcomes of meta-analyses.

Outcomes	Numbers of studies included	Sample sizes	*OR*	95% *CI*
**Total complications**	8	552,553	1.41	1.01–1.98
**Surgical complications**				
Wound complications	20	706,107	1.77	1.50–2.10
Prosthetic joint infection	14	234,937	1.84	1.52–2.24
Aseptic loosening	7	112,637	1.62	1.12–2.34
Dislocation	4	113,130	1.23	1.00–1.50
Revision	10	171,261	2.12	1.46–3.08
**Systemic complications**				
Myocardial infarction	7	2,350,799	2.14	0.89–5.17
Cardiac arrest	5	2,200,439	4.90	2.26–10.60
Cerebrovascular accident	6	2,349,988	2.22	1.01–4.85
Pneumonia	8	2,521,518	2.35	1.17–4.74
Acute renal insufficiency	10	2,235,826	2.01	1.48–2.73
Urinary tract infection	8	2,351,420	1.40	0.94–2.08
Deep venous thrombosis	7	2,350,609	1.54	0.83–2.86
Pulmonary embolism	6	2,317,273	1.29	0.60–2.79
Sepsis	5	2,317,563	4.35	1.35–14.00
**Mortality**				
Inpatient mortality	2	2,118,935	12.37	4.46–34.28
30-day mortality	4	114,840	0.88	0.68–1.13
**Persistent opioid consumption**	3	183,544	1.64	1.39–1.92

*OR*, Odds ratio; 95% *CI*, 95% confidence intervals.

## Surgical complications

### Wound complications

Wound complications were assessed in 13 cohort studies (10, 14, 15, 19, 20, 41, 45–48, 55–57) and seven case-control studies (16, 24, 34, 38, 39, 42, 58) involving 706,107 patients. Meta-analysis showed that smoking increased the risk of wound complications after THA and TKA (*OR* 1.77, 95% *CI* 1.50–2.10; *I^2 ^*= 85%; Table 2).

### Prosthetic joint infection

Data on prosthetic joint infection were extracted from eight cohort studies (10, 20, 41, 46–48, 56, 57) and six case-control studies (24, 34, 38, 39, 42, 58) involving 234,937 patients. Meta-analysis showed that patients who smoked were at higher risk of prosthetic joint infection than patients who did not (*OR* 1.84, 95% *CI* 1.52–2.24; *I*^2 ^= 63%; Table 2).

### Aseptic loosening

Aseptic loosening was assessed in six cohort studies (23, 45, 46, 48, 50, 57) and one case-control study (18), involving a combined total of 112,637 patients. Meta-analysis showed that smoking increased the risk of aseptic loosening after THA/TKA (*OR* 1.62, 95% *CI* 1.12–2.34; *I*^2 ^= 34%; Table 2).

### Dislocation

Three cohort studies (48, 52, 57) and one case-control study (50) involving 113,130 patients reported the incidence of dislocation after THA. Meta-analysis showed that there was no significant difference in the risk of dislocation between smoking or non-smoking patients (*OR* 1.23, 95% *CI* 1.00–1.50; *I*^2 ^=^ ^0%; Table 2).

### Revision

Seven cohort studies (11, 45–48, 56, 57) and three case-control studies (35, 49, 50) involving 171,261 patients reported the incidence of revision. Meta-analysis showed that risk of revision was significantly higher among smoking patients (*OR* 2.12, 95% *CI* 1.46–3.08; *I*^2 ^=^ ^90%; Table 2).

## Systemic complications

### Circulatory complications

#### Myocardial infarction

Myocardial infarction was reported in seven cohort studies involving 2,350,799 patients (10, 14, 15, 19, 20, 51, 55). Meta-analysis showed that the risk of myocardial infarction after THA/TKA was greater in smoking patients than non-smoking patients, although the difference was not significant (*OR* 2.14, 95% *CI* 0.89–5.17; *I^2 ^*= 99%; Table 2).

#### Cardiac arrest

Data on cardiac arrest were extracted from five cohort studies involving 2,200,439 patients (10, 14, 19, 20, 51). Meta-analysis showed that smoking was associated with significantly higher risk of cardiac arrest after THA or TKA (*OR* 4.90, 95% *CI* 2.26–10.60; *I*^2 ^= 93%; Table 2).

#### Cerebrovascular accident

A total of six cohort studies involving 2,349,988 patients assessed the risk of cerebrovascular accident after THA/TKA (10, 15, 19, 20, 51, 55). Meta-analysis showed that smoking was associated with significantly greater risk of cerebrovascular accident (*OR* 2.22, 95% *CI* 1.01–4.85; *I*^2 ^=^ ^98%; Table 2).

### Respiratory complications

Pneumonia was assessed in seven cohort studies (10, 15, 19, 20, 45, 51, 55) and one case-control study (36) involving 2,521,518 patients. Meta-analysis showed that smoking significantly increased risk of pneumonia after THA or TKA (*OR* 2.35, 95% *CI* 1.17–4.74; *I*^2 ^= 100%; Table 2).

### Urinary complications

#### Acute renal insufficiency

Seven cohort studies (10, 14, 19, 20, 46, 51, 55) and three case-control studies (21, 22, 33) involving 2,235,826 patients reported the incidence of acute renal insufficiency after THA or TKA. Meta-analysis showed smoking to be associated with significantly higher risk of acute renal insufficiency (*OR* 2.01, 95% *CI* 1.48–2.73; *I*^2 ^=^ ^97%; Table 2).

#### Urinary tract infection

Data on urinary tract infection was extracted from eight cohort studies involving 2,351,420 patients (10, 14, 15, 19, 20, 46, 51, 55). Meta-analysis showed that risk of urinary tract infection was not significantly different between patients who smoked or did not (*OR* 1.40, 95% *CI* 0.94–2.08; *I*^2 ^= 99%; Table 2).

### Venous thromboembolism

Seven cohort studies involving 2,350,609 patients (10, 15, 19, 20, 46, 51, 55) and six cohort studies involving 2,317,273 patients (10, 15, 19, 20, 46, 51) separately assessed the incidence of deep venous thrombosis (DVT) and pulmonary embolism (PE). Meta-analysis showed no significant difference between patients who smoked or did not in the case of either DVT (*OR* 1.54, 95% *CI* 0.83–2.86; *I*^2 ^= 99%; Table 2) or PE (*OR* 1.29, 95% *CI* 0.60–2.79; *I*^2 ^= 99%; Table 2).

### Sepsis

Sepsis was assessed in four cohort studies (10, 19, 20, 51) and one case-control study (37) involving 2,317,563 patients. Meta-analysis showed that smoking was associated with significantly higher risk of sepsis after THA or TKA (*OR* 4.35, 95% *CI* 1.35–14.00; *I*^2 ^= 99%; Table 2).

## Mortality

Data were separately extracted from two (19, 51) and four cohort studies (14, 19, 20, 55) on inpatient mortality (2,118,935 patients) and 30-day mortality (114,840 patients). Meta-analysis showed that smoking was associated with significantly higher inpatient mortality (*OR* 12.37, 95% *CI* 4.46–34.28; *I*^2 ^= 95%; Table 2), but it did not significantly increase risk of 30-day mortality (*OR* 0.88, 95% *CI* 0.68–1.13; *I*^2 ^= 0%; Table 2).

## Readmission

Two studies involving 5,409 patients reported that smoking patients were at elevated risk of 30-day readmission (20), but there was no difference in readmission at 90 days after surgery (54).

## Opioid consumption

Two cohort studies (15, 40) and two case-control studies (13, 44) involving 183,792 patients reported postoperative opioid consumption. One study showed that smokers consumed significantly more opioids than non-smokers immediately after THA as well as 90 days after the surgery (40). Meta-analysis of another three studies showed that smoking was associated with elevated incidence of persistent opioid consumption within 1 year after THA or TKA (*OR* 1.64, 95% *CI* 1.39–1.92; *I*^2 ^= 93%; Table 2).

## Patient-reported outcomes

A total of four cohort studies (15, 43, 46, 47) and one case-control study (12) involving 1,235,726 patients reported data on patient-reported outcomes based on different scales. In one study, smokers achieved significantly smaller improvements on the WOMAC and SF-12 PCS than non-smokers within 1 year after THA or TKA (43). Another study found that smokers had lower HHS than non-smokers at six months (47). A study of THA patients found that smoking was associated with two trajectories of OHS-assessed functional recovery within the first postoperative year: “slow start”, characterized by no initial improvement, followed later by improvement; and “late dip”, characterized by initial improvement but subsequent deterioration (12). Another study found that smokers had lower OHS/OKS after THA/TKA at 6-month follow-up (15) but not at follow-up longer than 1 year (46, 47).

## Subgroup analysis and meta-regression

**Figure 2:**
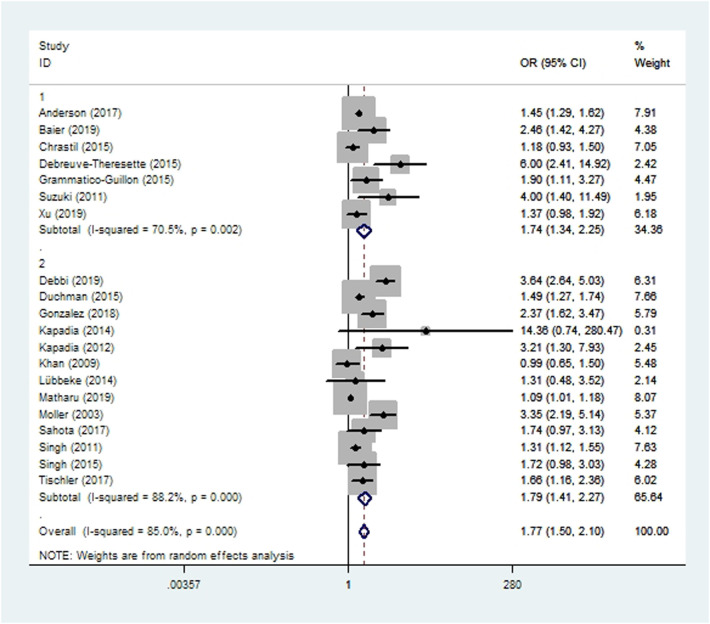
Forest plot of subgroup analysis for wound complications based on study type (1: case-control study; 2: cohort study).

**Figure 3:**
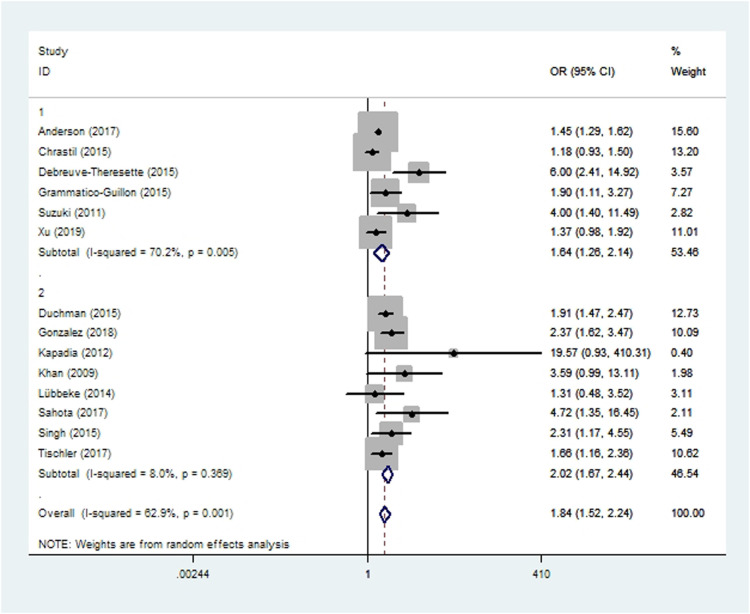
Forest plot of subgroup analysis for prosthetic joint infection based on study type (1: case-control study; 2: cohort study).

**Figure 4:**
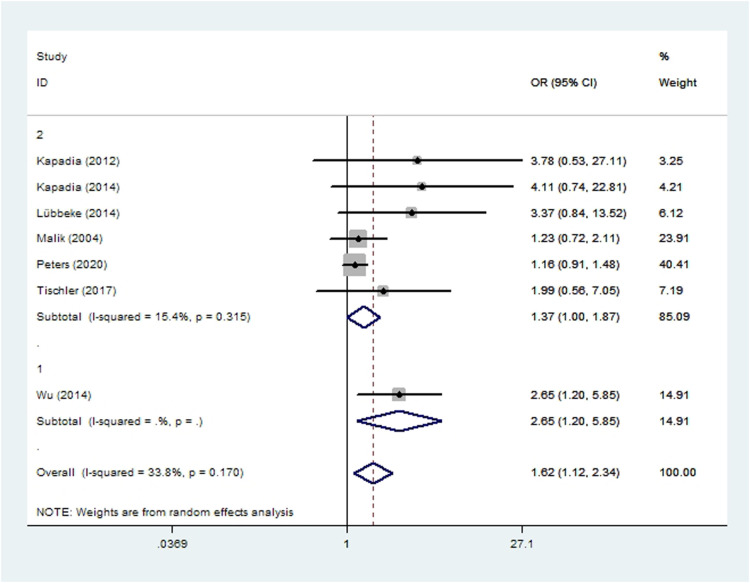
Forest plot of subgroup analysis for aseptic loosening based on study type (1: case-control study; 2: cohort study).

**Figure 5:**
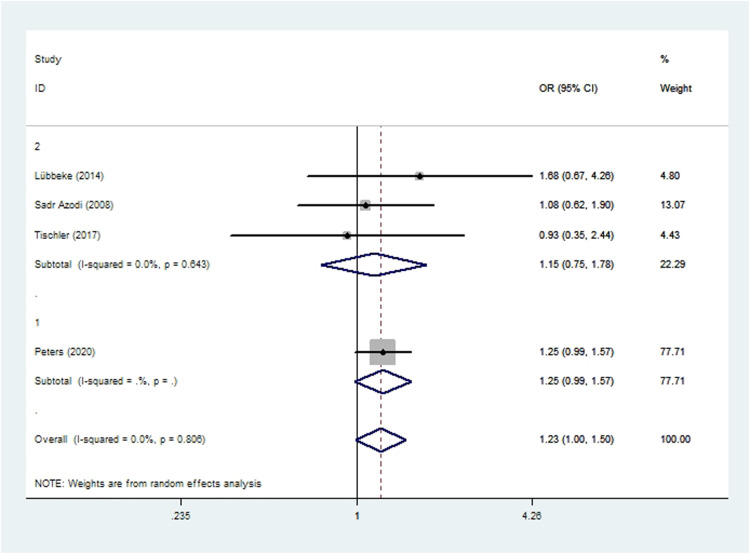
Forest plot of subgroup analysis for dislocation based on study type (1: case-control study; 2: cohort study).

**Figure 6:**
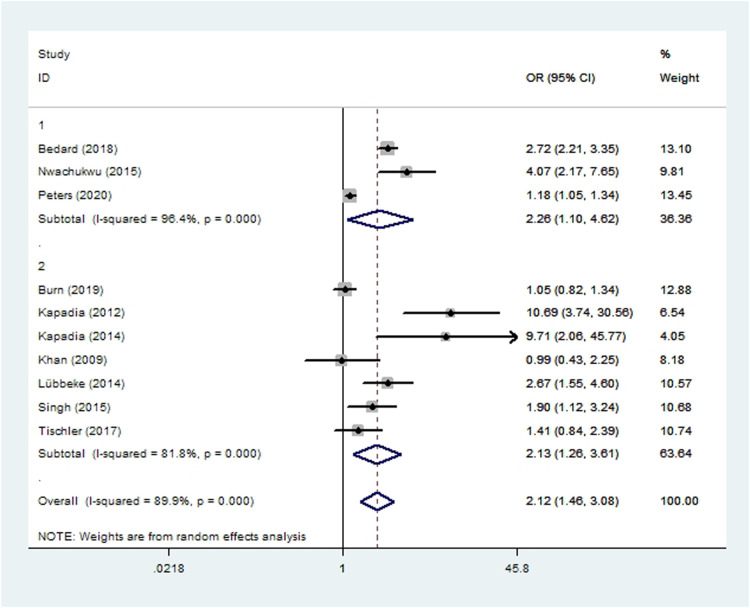
Forest plot of subgroup analysis for revision based on study type (1: case-control study; 2: cohort study).

**Figure 7:**
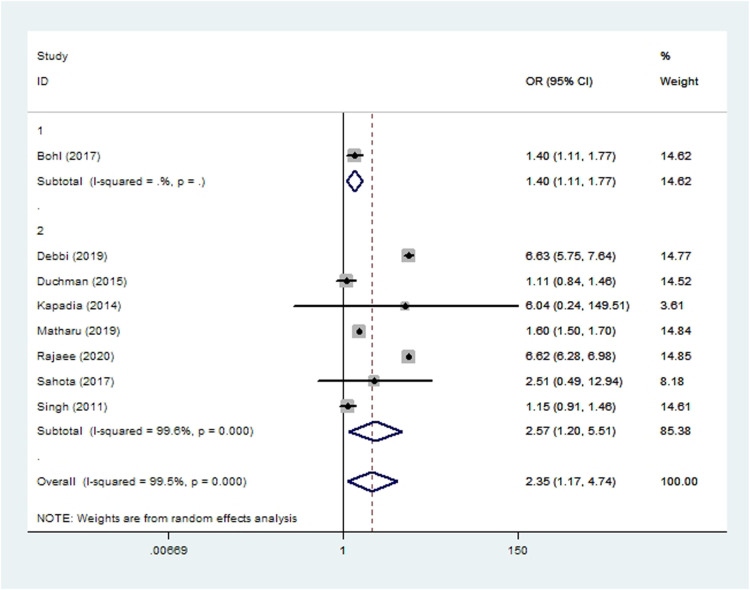
Forest plot of subgroup analysis for pneumonia based on study type (1: case-control study; 2: cohort study).

**Figure 8:**
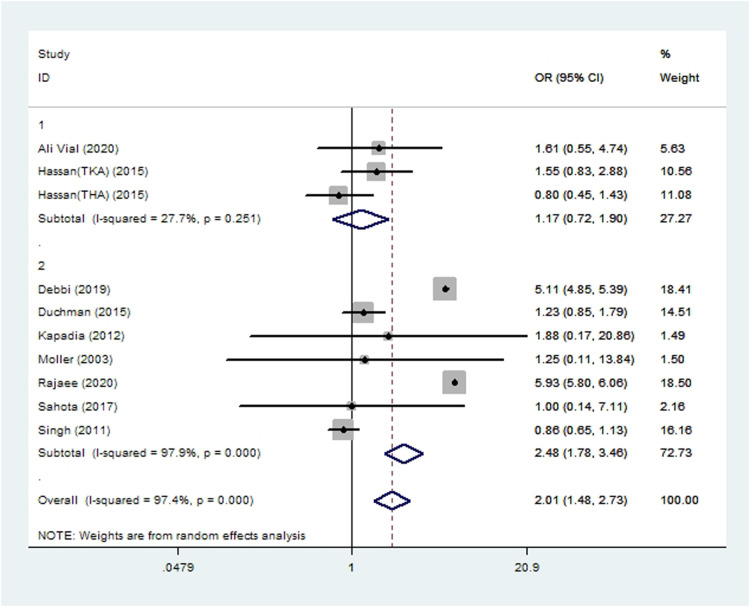
Forest plot of subgroup analysis for acute renal insufficiency based on study type (1: case-control study; 2: cohort study).

**Figure 9:**
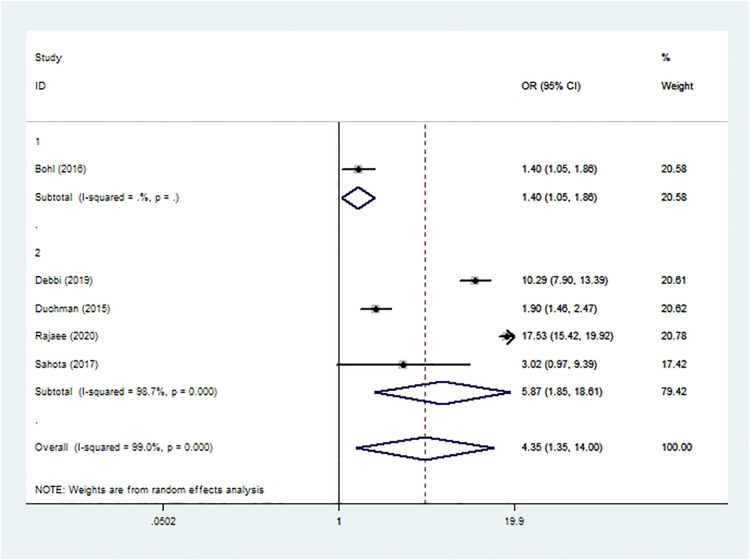
Forest plot of subgroup analysis for sepsis based on study type (1: case-control study; 2: cohort study).

**Figure 10:**
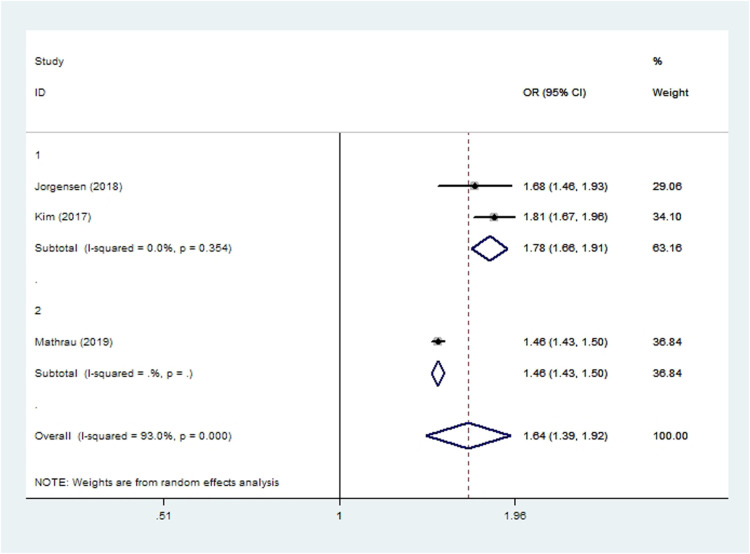
Forest plot of subgroup analysis for persistent opioid consumption based on study type (1: case-control study; 2: cohort study).

Subgroup analyses based on study type revealed no significant differences between cohort or case-control studies in incidences of wound complications, prosthetic joint infection, aseptic loosening, dislocation, revision, or persistent opioid consumption. Case-control studies were associated with significantly lower incidences of pneumonia, acute renal insufficiency and sepsis (Figures 2–10). Meta-regression analyses exploring the effects of potential sources of heterogeneity were conducted for the outcomes of wound complications, prosthetic joint infection, revision, and acute renal insufficiency, and significant subgroup effects were not found.

## Sensitivity analysis

Sensitivity analyses gave similar results as overall meta-analysis in the case of wound complications, prosthetic joint infection, dislocation, revision, pneumonia, urinary tract infection, DVT, PE, sepsis, 30-day mortality, and opioid consumption (Table 3). This suggests that these meta-analyses are likely to be robust and to represent true associations. In contrast, sensitivity analyses gave different results from the overall meta-analysis in the case of total complications, aseptic loosening, myocardial infarction, cardiac arrest, cerebrovascular accident, and acute renal insufficiency. These meta-analyses may therefore not be so robust.

**Table 3:** Results of sensitivity analysis.

Exclusion criterion	Studies	*OR*	95% *CI*	*I^2^*
**Total complications *OR:*1.41 (95% *CI*: 1.01-1.98), *I^2^*** **=** **99%**				
Studies causing heterogeneity	[14, 19, 20, 55]	1.18	1.11–1.27	42%
	[14, 19, 20, 45, 55]	1.17	1.13–1.21	0%
Studies with more than 100,000 patients	[15, 19]	1.23	1.03–1.47	85%
Studies with fewer than 1,000 patients	[14, 45]	1.26	0.88–1.82	100%
Studies with fewer than 100 events	[45]	1.37	0.97–1.92	99%
**Wound complications *OR*:1.77 (95% *CI*: 1.50-2.10), *I^2^*** **=** **85%**				
Studies causing heterogeneity	[14, 15, 19, 39]	1.53	1.36–1.73	48%
	[14-16, 19, 24, 39, 41]	1.42	1.30–1.55	19%
Studies with more than 100,000 patients	[15, 19]	1.70	1.47–1.98	67%
Studies with fewer than 1,000 patients	[14, 39, 45, 46, 58]	1.62	1.37–1.92	85%
Studies with NOS ≤ 6 points	[42, 47, 48]	1.85	1.54–2.22	87%
**Prosthetic joint infection *OR*:1.84 (95% *CI*: 1.52-2.24), *I^2^*** **=** **63%**				
Studies causing heterogeneity	[38, 39]	1.83	1.52–2.20	48%
	[34, 38, 39, 58]	2.03	1.72–2.40	3%
Studies with more than 50,000 patients	[10, 34]	2.05	1.55–2.73	64%
Studies with fewer than 1,000 patients	[39, 46, 58]	1.77	1.46–2.13	57%
Studies with NOS ≤ 6 points	[42, 47, 48]	1.85	1.49–2.29	69%
**Aseptic loosening *OR*:1.62 (95% *CI*: 1.12-2.34), *I^2^*** **=** **34%**				
Studies with fewer than 500 patients	[18, 23, 45]	1.56	0.91–2.69	26%
Studies with NOS ≤ 6 points	[18]	1.37	1.00–1.87	15%
**Dislocation *OR*:1.23 (95% *CI*: 1.00-1.50), *I^2^*** **=** **0%**				
Studies with more than 50,000 patients	[50]	1.15	0.75–1.78	0%
Studies with NOS ≤ 6 points	[48]	1.21	0.98–1.49	0%
**Revision *OR*:2.12 (95% *CI*: 1.46-3.08), *I^2^*** **=** **90%**				
Studies causing heterogeneity	[35, 45, 46, 48, 49]	1.19	1.04–1.36	14%
Studies with more than 50,000 patients	[50]	2.36	1.53–3.66	86%
Studies with fewer than 1,000 patients	[45, 46, 49]	1.59	1.11–2.28	90%
Studies with NOS ≤ 6 points	[47-49]	2.04	1.32–3.15	92%
**Myocardial infarction *OR*** **=** **2**.**14, 95% *CI*: 0.89-5.17, *I^2^*** **=** **99%**				
Studies causing heterogeneity	[19, 51]	1.11	0.85–1.46	45%
	[15, 19, 51]	0.95	0.74–1.22	0%
Studies with more than 1,000,000 patients	[51]	1.62	0.63–4.14	98%
Studies with fewer than 1,000 patients	[14]	2.11	0.83–5.32	99%
**Cardiac arrest *OR*** **=** **4.90, 95% *CI*: 2.26-20.93, *I*^2^** **=** **93%**				
Studies causing heterogeneity	[10]	7.65	6.70–8.73	0%
Studies with more than 1,000,000 patients	[51]	4.33	0.89–20.93	94%
Studies with fewer than 1,000 patients	[14]	4.64	2.07–10.38	95%
**Cerebrovascular accident *OR*** **=** **2.22, 95% *CI*: 1.01-4.85, *I*^2^** **=** **98%**				
Studies causing heterogeneity	[19, 51]	1.26	0.95–1.66	30%
	[15, 19, 51]	1.58	1.10–2.28	0%
Studies with more than 1,000,000 patients	[51]	1.79	0.91–3.53	93%
**Pneumonia *OR*** **=** **2.35, 95% *CI*: 1.17-4.74, *I*^2^** **=** **100%**				
Studies causing heterogeneity	[15, 19, 51]	1.24	1.07–1.43	0%
Studies with more than 1,000,000 patients	[51]	1.93	1.02–3.65	98%
Studies with fewer than 1,000 patients	[45]	2.27	1.11–4.63	100%
Studies with fewer than 1,00 events	[20, 45]	2.25	1.06–4.74	100%
**Acute renal insufficiency *OR*** **=** **2.01, 95% *CI*: 1.48-2.73, *I*^2^** **=** **97%**				
Studies causing heterogeneity	[19, 51]	1.02	0.84–1.23	0%
Studies with more than 1,000,000 patients	[51]	1.45	0.64–3.32	97%
Studies with fewer than 1,000 patients	[14, 21, 22, 33, 46]	2.53	1.80–3.55	99%
Studies with fewer than 1,00 events	[14, 20-22, 33, 46]	2.60	1.84–3.65	99%
**Urinary tract infection *OR*** **=** **1.40, 95% *CI*: 0.94-2.08, *I*^2^** **=** **99%**				
Studies causing heterogeneity	[19, 51]	0.93	0.85–1.02	20%
Studies with more than 1,000,000 patients	[51]	1.25	0.73–2.12	99%
Studies with fewer than 1,000 patients	[14, 46]	1.37	0.89–2.12	99%
Studies with fewer than 1,00 events	[14, 20, 46]	1.42	0.89–2.26	100%
Deep venous thrombosis *OR* = 1.54, 95% *CI*: 0.83-2.86, *I*^2^ = 99%				
Studies causing heterogeneity	[19, 51]	0.96	0.89–1/03	0%
Studies with more than 1,000,000 patients	[51]	1.32	0.83–2.11	95%
Studies with fewer than 1,000 patients	[46]	1.53	0.81–2.88	99%
Studies with fewer than 1,00 events	[20, 46]	1.59	0.81–3.13	99%
**Pulmonary embolism *OR*** **=** **1.29, 95% *CI*: 0.60-2.79, *I*^2^** **=** **99%**				
Studies causing heterogeneity	[51]	1.01	0.91–1.13	0%
Studies with more than 1,000,000 patients	[51]	1.01	0.91–1.13	0%
Studies with fewer than 1,000 patients	[46]	1.29	0.59–2.85	99%
**Sepsis *OR*** **=** **4.35, 95% *CI*: 1.35-14.00, *I*^2^** **=** **99%**				
Studies causing heterogeneity	[19, 51]	1.70	1.28–2.26	41%
	[19, 37, 51]	1.94	1.51–2.51	0%
Studies with more than 1,000,000 patients	[51]	3.02	1.04–8.74	98%
Studies with fewer than 1,00 events	[20]	4.70	1.29–17.14	99%
**30-day mortality *OR*** **=** **0.88, 95% *CI*: 0.68-1.13, *I*^2^** **=** **0%**				
Studies with fewer than 1,000 patients	[14]	0.87	0.68–1.12	0%
Studies with fewer than 100 events	[14, 20]	0.87	0.68–1.12	0%
**Opioid consumption *OR*** **=** **1.64, 95% *CI*: 1.39-1.92, *I*^2^** **=** **93%**				
Studies causing heterogeneity	[15]	1.78	1.66–1.91	0%

NOS, Newcastle-Ottawa scale.

## Quality of evidence

The quality of evidence according to the GRADE system was low or very low for all outcomes (Supplementary Table S1).

## Discussion

A systematic review of studies published up to 2010 reported an association between smoking and composite risk of any postoperative complication or death (25), but numerous studies published since then have suggested that smoking exerts more complex effects on outcomes after THA or TKA. Therefore, we conducted the present review and meta-analysis to gain a comprehensive understanding based on all available evidence.

Smoking interferes with all phases of wound healing, including hemostasis, wound contraction, proliferation and remodeling (59), and this has been observed following many types of surgery (7, 59, 60). In addition, recent reports have described a possible correlation between smoking and prosthetic joint infection. The results of the present meta-analysis show that smoking is associated with higher incidences of wound complications and prosthetic joint infection, and the similar results obtained in all sensitivity analyses suggests that our findings are robust.

Smoking also has deleterious effects on bone metabolism (61, 62). It exerts toxic effects directly on bone cells and indirectly by affecting hormones, vitamin D, and oxygenation; it inhibits bone formation and accelerates bone absorption (61–63). Since smoking causes bone loss, it may be associated with periprosthetic osteolysis and subsequent aseptic loosening. After total joint arthroplasy, smokers show significantly lower serum levels of osteogenic markers than non-smokers, suggesting that smoking affects bone formation (61); whether the same is true for aseptic loosening is unclear (18, 23, 64, 65). The present meta-analysis supports the idea that smoking increases the risk of aseptic loosening after THA and TKA, but the different results obtained from sensitivity analyses suggest the need to verify these findings in future work. Since prosthetic joint infection and aseptic loosening are common causes for revision after THA and TKA (66, 67), the ability of smoking to increase the risk of these two complications may explain why it increased the risk of revision in our meta-analysis.

Our study highlights associations between smoking and significantly elevated risk of pneumonia and sepsis after THA and TKA. Smoking can increase the incidence of community-acquired pneumonia by impairing mucociliary clearance and increasing bacterial adherence (68, 69); as well as by causing changes in cellular and humoral immune system function (70). Smoking patients may therefore be at higher risk of postoperative pneumonia due to structural mechanisms and systemic immune dysfunction, which may help explain the results of our meta-analysis. Sepsis following total joint arthroplasty can prove devastating: it significantly increases risk of mortality as well as healthcare costs (37). Surgical site infection and pneumonia are the common sources of sepsis after THA and TKA (37), and the ability of smoking to increase the risk of both types of infection may explain its association with elevated risk of sepsis in our meta-analysis.

Smoking contributes significantly to nearly all cardio-cerebrovascular morbidity and mortality, ranging from chronic diseases of hypertension to acute clinical events such as myocardial infarction and cardiac arrest (71). The primary cause of cardio-cerebrovascular dysfunction appears to be oxidative stress caused by smoke exposure (72). Consistent with this literature, our meta-analysis shows that smoking is associated with significantly higher risk of postoperative cardiac arrest and cerebrovascular accident. In addition, we found that patients who smoke are also more likely to experience myocardial infarction after THA and TKA, although the difference in risk is not significant.

Acute renal insufficiency and urinary tract infection are the most common urinary complications after THA and TKA. In smokers, the genitourinary system is directly exposed to tobacco toxins that are excreted in urine, and the system may also be affected by systemic immune dysfunction caused by smoking (3). Our meta-analysis suggests that smoking is associated with elevated risk of acute renal insufficiency, but not of urinary tract infection, after THA or TKA.

In addition to complications, patient management after total joint arthroplasty aims to optimize the subjective feelings of patients, so patient-reported outcomes are increasingly used in clinical settings (43). Although we were able to conduct only qualitative synthesis, the outcomes of included studies are consistent: smoking is associated with worse patient-reported outcomes during the first year after THA and TKA. The association between smoking and elevated postoperative opioid consumption after these surgeries is an interesting finding. Nicotine in tobacco modulates pain perception and the body's natural neuroendocrine opioid system: it partly counteracts the analgesic effects of opioid medications and increases pain sensation, leading to greater opioid consumption and even dependence (73).

The strengths of this meta-analysis include the extensive literature searching, inclusion of a large amount of updated literature, and comprehensive investigation of the association between smoking and various postoperative complications and clinical outcomes. Moreover, we conducted subgroup analyses, meta-regression, sensitivity analyses, and GRADE evaluation of the evidence. On the other hand, our study also has several limitations. First, as the definitions of smoking status in most included studies were unclear or ambiguous, we could not evaluate possible differences in outcomes between current or previous smokers, between patients with long or short smoking histories, or between patients who smoke fewer or more cigarettes per day. Second, due to the lack of available data, we were unable to assess whether smoking shows a dose-response association with complications. Third, the unexplained heterogeneity and the generally low level of quality of evidence for outcomes made the conclusions less robust.

Despite these limitations, this systematic review and meta-analysis provide an up-to-date overview of the impact of smoking on clinical outcomes after TKA or THA. We find that smoking is associated with increased risks of numerous complications, inpatient mortality, persistent opioid consumption, and worse 1-year patient-reported outcomes. These findings suggest clinicians to make every effort to persuade THA/TKA patients to quit smoking before surgery, and pre-surgical protocols and perioperative managements should give special consideration to smoking patients undergoing THA or TKA.

## Data Availability

The original contributions presented in the study are included in the article/Supplementary Material, further inquiries can be directed to the corresponding author/s.
