# Supplementary material for: Associations between smoking and clinical outcomes after total hip and knee arthroplasty: A systematic review and meta-analysis
Source: Front Surg. 2022 Nov 2;9:970537. doi: 10.3389/fsurg.2022.970537 (PMC9666709; doi:10.3389/fsurg.2022.970537)
Supplement: Supplementary file 1 [file Table4.doc]

**Supplemental Table 1: Summarized outcomes in Grading of Recommendations Assessment, Development, and Evaluation (GRADE)**

| **Clinical outcomes after THA or TKA among smoking patients versus non-smoking patients** | | | | | | |
| --- | --- | --- | --- | --- | --- | --- |
| **Patient or population:** Patients with THA and TKA **Settings:**  **Intervention:** Smoking | | | | | | |
| **Outcome** | **Illustrative comparative risks* (95% CI)** | | **Relative effect (95% CI)** | **No. of Participants (studies)** | **Quality of evidence (GRADE)** | **Comments** |
|  | Assumed risk | Corresponding risk |  |  |  |  |
|  | **Control** | **Smoking** |  |  |  |  |
| **Total complications** | **Study population** | | **OR 1.41**  (1.01 to 1.98) | 552553 (8 studies) | ⊕⊝⊝⊝ **very low**^1,2^ |  |
|  | **56 per 1000** | **77 per 1000** (57 to 105) |  |  |  |  |
|  | **Moderate** | |  |  |  |  |
|  | **87 per 1000** | **118 per 1000** (88 to 159) |  |  |  |  |
| **Wound complications** | **Study population** | | **OR 1.77**  (1.5 to 2.1) | 706107 (20 studies) | ⊕⊝⊝⊝ **very low**^2,3^ |  |
|  | **10 per 1000** | **17 per 1000** (15 to 20) |  |  |  |  |
|  | **Moderate** | |  |  |  |  |
|  | **17 per 1000** | **30 per 1000** (25 to 35) |  |  |  |  |
| **Prosthetic joint infection** | **Study population** | | **OR 1.84**  (1.52 to 2.24) | 234937 (14 studies) | ⊕⊝⊝⊝ **very low**^2,3^ |  |
|  | **14 per 1000** | **25 per 1000** (21 to 30) |  |  |  |  |
|  | **Moderate** | |  |  |  |  |
|  | **8 per 1000** | **15 per 1000** (12 to 18) |  |  |  |  |
| **Aseptic loosening** | **Study population** | | **OR 1.62**  (1.12 to 2.34) | 112637 (7 studies) | ⊕⊕⊝⊝ **low** |  |
|  | **7 per 1000** | **11 per 1000** (7 to 15) |  |  |  |  |
|  | **Moderate** | |  |  |  |  |
|  | **6 per 1000** | **10 per 1000** (7 to 14) |  |  |  |  |
| **Dislocation** | **Study population** | | **OR 1.23**  (1 to 1.5) | 113130 (4 studies) | ⊕⊕⊝⊝ **low** |  |
|  | **6 per 1000** | **8 per 1000** (6 to 9) |  |  |  |  |
|  | **Moderate** | |  |  |  |  |
|  | **7 per 1000** | **9 per 1000** (7 to 10) |  |  |  |  |
| **Revision** | **Study population** | | **OR 2.12**  (1.46 to 3.08) | 171261 (10 studies) | ⊕⊝⊝⊝ **very low**^1,2,3,4^ |  |
|  | **21 per 1000** | **44 per 1000** (31 to 63) |  |  |  |  |
|  | **Moderate** | |  |  |  |  |
|  | **16 per 1000** | **33 per 1000** (23 to 48) |  |  |  |  |
| **Myocardial infarction** | **Study population** | | **OR 2.14**  (0.89 to 5.17) | 2350799 (7 studies) | ⊕⊕⊝⊝ **low**^1,2,4^ |  |
|  | **1 per 1000** | **2 per 1000** (1 to 4) |  |  |  |  |
|  | **Moderate** | |  |  |  |  |
|  | **3 per 1000** | **6 per 1000** (3 to 15) |  |  |  |  |
| **Cardiac arrest** | **Study population** | | **OR 4.9**  (2.26 to 10.6) | 2200439 (5 studies) | ⊕⊝⊝⊝ **very low**^4,5^ |  |
|  | **0 per 1000** | **1 per 1000** (1 to 2) |  |  |  |  |
|  | **Moderate** | |  |  |  |  |
|  | **0 per 1000** | **0 per 1000** (0 to 0) |  |  |  |  |
| **Cerebrovascular accident** | **Study population** | | **OR 2.22**  (1.01 to 4.85) | 2349988 (6 studies) | ⊕⊕⊝⊝ **low**^2,4^ |  |
|  | **0 per 1000** | **1 per 1000** (0 to 2) |  |  |  |  |
|  | **Moderate** | |  |  |  |  |
|  | **1 per 1000** | **2 per 1000** (1 to 5) |  |  |  |  |
| **Pneumonia** | **Study population** | | **OR 2.35**  (1.17 to 4.74) | 2521518 (8 studies) | ⊕⊕⊝⊝ **low**^2,4^ |  |
|  | **3 per 1000** | **6 per 1000** (3 to 12) |  |  |  |  |
|  | **Moderate** | |  |  |  |  |
|  | **3 per 1000** | **7 per 1000** (4 to 14) |  |  |  |  |
| **Readmission** | See comment | See comment | Not estimable | 5409 (2 studies) | See comment | Smoking patients were at elevated risk of 30-day readmission, but there was no difference in readmission at 90 days after surgery. |
| **Acute renal insufficiency** | **Study population** | | **OR 2.01**  (1.48 to 2.73) | 2235826 (10 studies) | ⊕⊝⊝⊝ **very low**^1,2,3,4^ |  |
|  | **10 per 1000** | **19 per 1000** (14 to 26) |  |  |  |  |
|  | **Moderate** | |  |  |  |  |
|  | **8 per 1000** | **16 per 1000** (12 to 22) |  |  |  |  |
| **Opioid consumption** | **Study population** | | **OR 1.64**  (1.39 to 1.92) | 183544 (3 studies) | ⊕⊝⊝⊝ **very low**^2^ |  |
|  | **192 per 1000** | **280 per 1000** (248 to 313) |  |  |  |  |
|  | **Moderate** | |  |  |  |  |
|  | **147 per 1000** | **220 per 1000** (193 to 249) |  |  |  |  |
| **Urinary tract infection** | **Study population** | | **OR 1.4**  (0.94 to 2.08) | 2351420 (8 studies) | ⊕⊝⊝⊝ **very low**^1,2^ |  |
|  | **14 per 1000** | **19 per 1000** (13 to 28) |  |  |  |  |
|  | **Moderate** | |  |  |  |  |
|  | **13 per 1000** | **18 per 1000** (12 to 27) |  |  |  |  |
| **Deep venous thrombosis** | **Study population** | | **OR 1.54**  (0.83 to 2.86) | 2350609 (7 studies) | ⊕⊝⊝⊝ **very low**^1,2^ |  |
|  | **3 per 1000** | **4 per 1000** (2 to 8) |  |  |  |  |
|  | **Moderate** | |  |  |  |  |
|  | **6 per 1000** | **9 per 1000** (5 to 17) |  |  |  |  |
| **Pulmonary embolism** | **Study population** | | **OR 1.29**  (0.6 to 2.79) | 2317273 (6 studies) | ⊕⊝⊝⊝ **very low**^1,2^ |  |
|  | **2 per 1000** | **3 per 1000** (1 to 6) |  |  |  |  |
|  | **Moderate** | |  |  |  |  |
|  | **4 per 1000** | **5 per 1000** (2 to 11) |  |  |  |  |
| **Sepsis** | **Study population** | | **OR 4.35**  (1.35 to 14) | 2317563 (5 studies) | ⊕⊝⊝⊝ **very low**^2,4,5^ |  |
|  | **0 per 1000** | **2 per 1000** (1 to 7) |  |  |  |  |
|  | **Moderate** | |  |  |  |  |
|  | **3 per 1000** | **13 per 1000** (4 to 40) |  |  |  |  |
| **Inpatient mortality** | **Study population** | | **OR 12.37**  (4.46 to 34.28) | 2118935 (2 studies) | ⊕⊕⊝⊝ **low**^2,5,6^ |  |
|  | **0 per 1000** | **2 per 1000** (1 to 6) |  |  |  |  |
|  | **Moderate** | |  |  |  |  |
|  | **0 per 1000** | **0 per 1000** (0 to 0) |  |  |  |  |
| **30-day mortality** | **Study population** | | **OR 0.88**  (0.68 to 1.13) | 114840 (4 studies) | ⊕⊕⊝⊝ **low** |  |
|  | **3 per 1000** | **2 per 1000** (2 to 3) |  |  |  |  |
|  | **Moderate** | |  |  |  |  |
|  | **2 per 1000** | **2 per 1000** (1 to 2) |  |  |  |  |
| **Patient-reported outcomes** | See comment | See comment | Not estimable | 1235105 (4 studies) | See comment | Data not presented in a format to allow meta-analysis. Smoking was associated with worse patient-reported outcomes |
| *The basis for the **assumed risk** (e.g. the median control group risk across studies) is provided in the footnotes. The **corresponding risk** (and its 95% confidence interval) is based on the assumed risk in the comparison group and the **relative effect** of the intervention (and its 95% CI).  **CI:** Confidence interval; **OR:** Odds ratio; | | | | | | |
| GRADE Working Group grades of evidence **High quality:** Further research is very unlikely to change our confidence in the estimate of effect.  **Moderate quality:** Further research is likely to have an important impact on our confidence in the estimate of effect and may change the estimate. **Low quality:** Further research is very likely to have an important impact on our confidence in the estimate of effect and is likely to change the estimate. **Very low quality:** We are very uncertain about the estimate. | | | | | | |
| ^1^ Inconsistent results across studies  ^2^ Unexplained heterogeneity  ^3^ Evaluation of funnel plots and Harbord tests showed a certain level of publication bias ^4^ OR > 2 ^5^ Studies included relatively few events and thus had wide confidence intervals around the effect estimate ^6^ OR > 5 | | | | | | |
